# The Effect of Botulinum Toxin A on Ischemia-Reperfusion Injury in a Rat Model

**DOI:** 10.1155/2017/1074178

**Published:** 2017-05-15

**Authors:** Tae Hwan Park, Yun Joo Park

**Affiliations:** ^1^Department of Plastic and Reconstructive Surgery, CHA Bundang Medical Center, CHA University, 59 Yatap-ro, Bundang-gu, Seongnam, Gyeonggi-do 13496, Republic of Korea; ^2^Department of Radiology, Hallym University College of Medicine and Hallym University Sacred Heart Hospital, No. 22, Gwanpyeong-ro 170beon-gil, Dongan-gu, Anyang, Gyeonggi-do 14068, Republic of Korea

## Abstract

**Introduction:**

While studies using various materials to overcome ischemia-reperfusion (IR) injury are becoming increasingly common, studies on the effects of botulinum toxin A (BoTA) on IR injury in musculocutaneous flaps are still limited. The purpose of this study was to examine our hypotheses that BoTA provide protection of musculocutaneous flap from ischemia-reperfusion injury.

**Method:**

Five days after pretreatment injection (BoTA versus normal saline), a right superior musculocutaneous flap (6 × 1.5 cm in size) was made. Ischemia was created by a tourniquet strictly wrapping the pedicle containing skin and muscle for 8 h. After ischemia, the tourniquet was cut, and the musculocutaneous flap was reperfused.

**Results:**

The overall survival percentage of flap after 8 h of pedicle clamping followed by reperfusion was 87.32 ± 3.67% in the control group versus 95.64 ± 3.25% in the BoTA group (*p* < 0.001). The BoTA group had higher expression of CD34, HIF-1*α*, VEGF, and NF-kB comparing to control group in qRT-PCR analysis.

**Conclusions:**

In this study, we found that local BoTA preconditioning yielded significant protection against IR injury in a rat musculocutaneous flap model.

## 1. Introduction 

Ischemia-reperfusion injury occurs when circulation is abruptly restored following prolonged ischemia and it is well-known that high levels of calcium and tissue neutrophil accumulation cause cellular damage and produce reactive oxygen species (ROS) during reperfusion and trigger IR injury [1]. Many studies have tried to discover pharmacologic or surgical interventions that may alleviate IR flap injuries [2–13]. Although several approaches gained popularity based on promising experimental results, their clinical applications remain extremely rare owing to their limited safety. On the other hand, botulinum toxin A is considered safe and is currently used clinically in a wide variety of plastic and reconstructive surgery fields [14]. In addition, recent studies have shown that BoTA increases skin flap survival via various mechanisms [15–19]. The toxin appears to not only have a positive effect on muscle circulation and vessels [20–23] but also apparently have the ability to alleviate IR injury in muscle flaps [6, 24]. The purpose of this study was to examine our hypotheses that BoTA provide protection of musculocutaneous flap from ischemia-reperfusion injury.

## 2. Materials and Methods 

### 2.1. Experiment Model and Flap Design

All animal protocols used in this study were approved by the Institutional Animal Care and Use Committee. Twenty-four male Sprague–Dawley rats weighing 240–280 g and aged 7 weeks were housed individually in an animal resources facility in a room at a controlled temperature (20–22.8°C) and under a 12 h light/dark cycle. They were provided with food and water ad libitum. The rats were randomly assigned to 2 groups: the BoTA group (*n* = 12) or the control group (*n* = 12).

**Figure 1:**
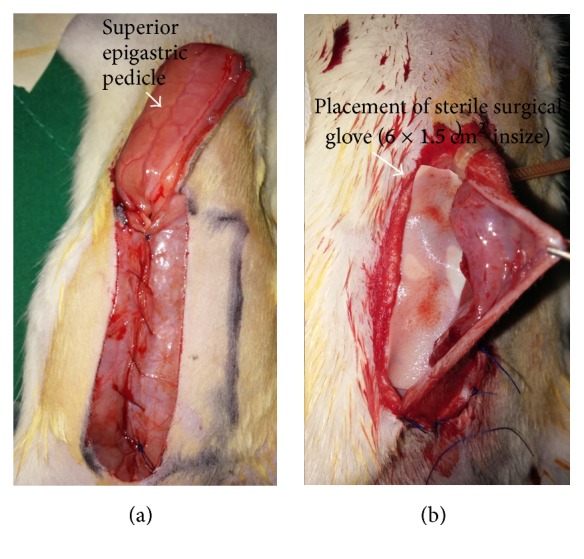
A harvesting of right superior musculocutaneous flap. Flap (6 × 1.5 cm in size) was made after the inferior origin was detached at the caudal border. Any musculofascial defect in the abdominal wall was closed primarily using Vicryl 4-0 (a). To prevent revascularization of the overlying flap, a 6 × 1.5 cm of tailored sterile surgical glove was placed under the flap following applying a tourniquet that tightly wrapped the pedicle containing muscle and skin (b).

Using a chamber, the animals underwent general anesthesia with 5% isoflurane (Aerane®; Ilsung Pharmaceuticals, Seoul, Korea) initially, and anesthesia was maintained with 1.5% isoflurane through a nasal cone until the end of the procedure. After shaving the ventral hair, a rectangular-shaped flap was marked. Rectangular flap (6 × 1.5 cm) was created on the right hemiabdomen of each rat using the costal margins as the superior border (Figure 1). Five days prior to flap elevation, vials of lyophilized BoTA were reconstituted in 10 mL of normal saline solution in a 100 international units' (IU) vial to a final concentration of 10 IU/mL. The BoTA group (*n* = 12) was pretreated with subdermal injections of 9 IU of BoTA (BOTOX®) (Allergan, Irvine, CA, USA) distributed evenly across 3 zones of the proximal, middle, and distal areas of the flap (0.3 mL each). The control group (*n* = 12) was pretreated with 0.9 mL of normal saline.

**Figure 2:**
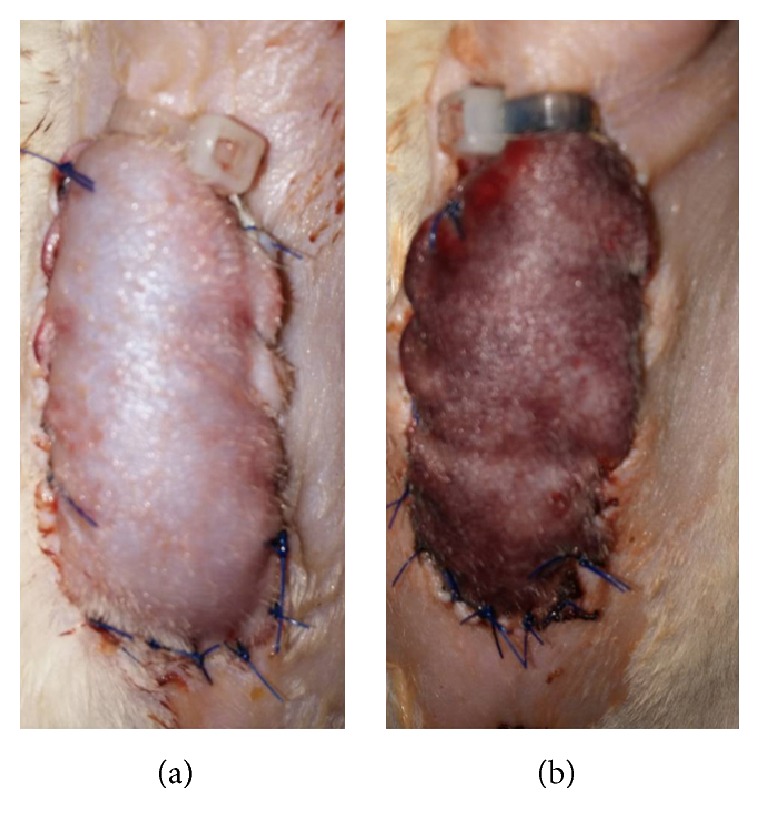
Representative gross findings created by a tourniquet strictly wrapping the pedicle containing skin and muscle. (a) Immediately after ischemia creation; (b) 8 hrs after ischemia creation.

Five days after pretreatment injection, a plastic surgeon who was blinded to the injected material made a 6 cm vertical midline incision (a depth just above the posterior rectus sheath). The musculocutaneous flap was elevated from the midline toward the right lateral side, carefully preserving all musculocutaneous perforators originating from the right rectus abdominis muscle. A right superior musculocutaneous flap (6 × 1.5 cm in size) was made after the inferior origin was detached at the caudal border. Any musculofascial defect in the abdominal wall was closed primarily using PDS 4-0 (Figure 1(a)). To prevent revascularization of the overlying flap, a 6 × 1.5 cm of tailored sterile surgical glove was placed under the flap, and to achieve appropriate ischemia we used a plastic tourniquet (PK-150 MF, Cable tie, GongRyongUhang, South Korea) that tightly wrapped the rectus muscle containing pedicle for 8 h (Figure 1(b)). The skin paddle was returned to its bed using 4-0 nylon. After 8 h of ischemia, a physician blinded to the injected material cut off the tourniquet to permit reperfusion (Figure 2).

### 2.2. Gross Evaluation

The flap survival was evaluated on the five postoperative day. On that day, a digital photograph was taken, and the survival percentage of flap was calculated using a transparent sheet and ImageJ® software (National Institutes of Health, Bethesda, MD). The survival percentage of flap was assessed independently by 2 investigators who were blinded to the treatment groups. When the survival area was not definite through the external skin, inner side of the flap was checked to clearly evaluate the survival area. The survival percentage of flap was expressed as a percentage of the total flap area (survival percentage of flap [%] = [viable area/total area] × 100) and used to determine the statistical significance of the differences between groups.

### 2.3. Molecular Evaluation

After 8 h of ischemia, a physician blinded to the injected material cut off the tourniquet to permit reperfusion. Sixteen hours after reperfusion, whole tissue specimens from each area were immediately snap-frozen in liquid nitrogen and then stored at −80°C. Analysis of samples began with quantitative real time polymerase chain reaction (qRT-PCR) of CD34, hypoxia inducible factor 1 alpha (HIF-1*α*), nuclear factor-kappaB (NF-kB), and vascular endothelial growth factor (VEGF) using primers for each gene, while glyceraldehyde-3-phosphate dehydrogenase (GAPDH) was used as a housekeeping gene control. The results were evaluated using Light Cycler® 480 analysis software. All of the reactions were performed in triplicate. Each gene expression of both groups was evaluated as a fold change relative to the gene expression of the proximal area of control group.

### 2.4. Statistical Analysis

The results of the experiments are expressed as mean ± SD. For comparison of the survival percentage of flap, Student's* t*-test was used. A *p* value < 0.05 was considered statistically significant.

## 3. Results 

### 3.1. Gross Evaluation

**Figure 3:**
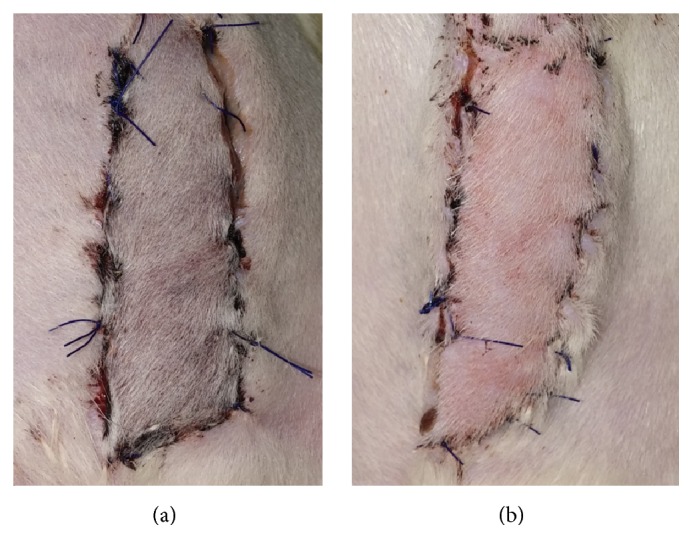
Representative gross findings of the musculocutaneous flaps in representative animals of the BoTA (a) and control (b) group. The BoTA group showed nearly complete survival of the flap whereas control group revealed partial necrosis of the flap.

The overall survival percentage was 87.32 ± 3.67% in the control group versus 95.64 ± 3.25% in the BoTA group (*p* < 0.001). The representative gross images are presented in Figure 3.

### 3.2. Molecular Evaluation

**Figure 4:**
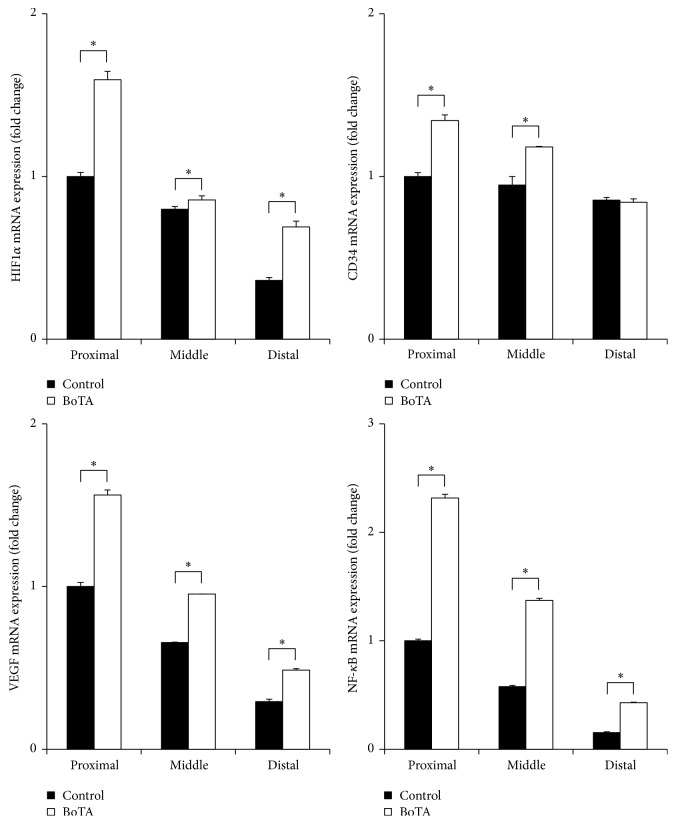
*Effects of BoTA on the Gene Expression of Muscle Tissue.* Relative mRNA expression of CD34, HIF1*α*, NF-kB, and VEGF by qRT-PCR of the rectus muscle considering each gene expression of proximal area in control group as 1. Gene expression of the rectus muscle was higher in the BoTA group than in the control group with statistical significance except CD34 expression in distal area. ^*∗*^*p* < 0.05.

**Figure 5:**
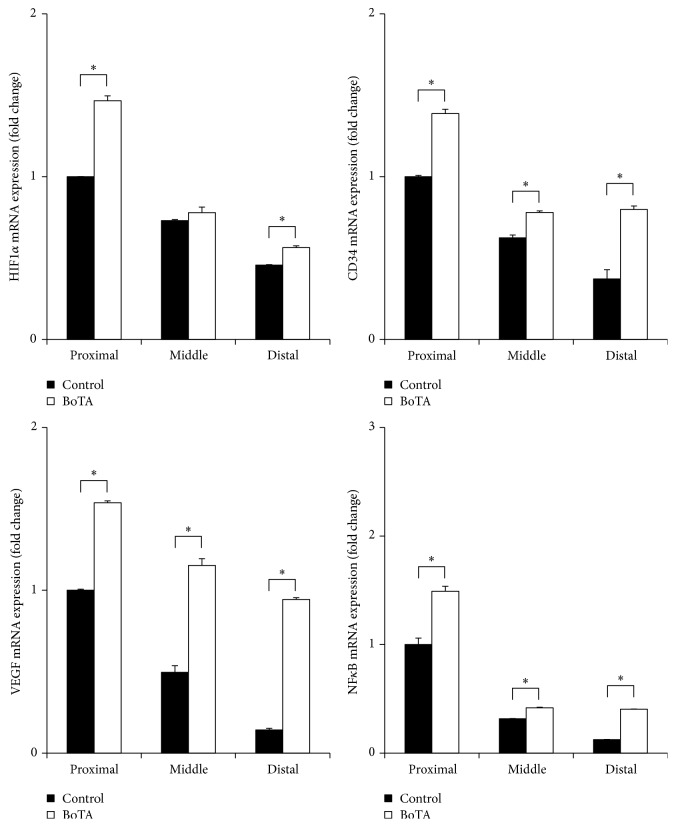
*Effects of BoTA on the Gene Expression of Skin Tissue.* Relative mRNA expression of CD34, HIF1*α*, NF-kB, and VEGF by qRT-PCR of the skin considering each gene expression of proximal area in control group as 1. Gene expression of the skin was higher in the BoTA group than in the control group with statistical significance except HIF1*α* expression in middle area. ^*∗*^*p* < 0.05.

Regarding skin, the relative mRNA expression of* CD34, HIF-1α, NF-kB,* and* VEGF* was higher in the BoTA group than the control group in each area. In muscle tissue, the relative mRNA expression of* CD34, HIF-1α, NF-kB,* and* VEGF* was higher in the BoTA group than the control group in each area except* CD34 *expression in proximal area where the difference was not significant (0.0657 ± 0.0013 versus 0.00647 ± 0.0016, *p* = 0.431). The relative mRNA expression level of each gene is shown in Figures 4 and 5.

## 4. Discussion 

In this study, we used a rat model of a musculocutaneous flap to demonstrate the efficacy of local BoTA preconditioning for protecting skin and muscle against IR injury at both the gross and molecular level. According to our literature review, only two studies have shown the protective effects of BoTA against IR injury of muscle tissue [6, 24]. One study by Kucuker et al. showed BoTA's chemical denervation effect on the biceps femoris muscle, and another study by Akcal et al. showed a protective effect following perivascular or intramuscular (right gastrocnemius muscle) BoTA injection on muscle flaps in an IR injury model. However, to the best of our knowledge, there has been no study on the protective effect of local BoTA pretreatment against IR injury in musculocutaneous flaps.

Recently, BoTA has been used in experimental studies and noted for its positive effects on flap survival [15–18, 25–27]. Exactly what mechanism is involved in increasing flap viability has not yet been clearly elucidated, but there are several intriguing hypotheses. One of the suggested mechanisms is direct vasodilatation [15]. In another study, sympathectomy was suggested as a possibility [16]. Kucuker et al. studied BoTA's protective effect in reperfused muscle and demonstrated that chemical denervation with BoTA was equal to surgically denervating muscles to protect against IR injury. This was a result of increased muscle ischemia tolerance [6]. In another study, it was hypothesized that BoTA may have a role in preconditioning through the release of substance P, calcitonin gene-related peptide (CGRP), and VEGF [24]. In 2007, Matic et al. showed increased glucose uptake and blood flow after BoTA injections [22]. In another recent study, Welham et al. reported that superoxide dismutase and energy metabolism can increase after BoTA injection [28]. It has also been shown that BoTA administration can increase CD31 and inducible nitric oxide synthase expression in skin flaps [16].

In our study, we injected BoTA 5 days before surgery to achieve maximum activity, as defined in a previous study [6, 15]. We evaluated the HIF-1*α* expression levels in both skin and muscle tissues. A protein that is found in mammal cells growing under hypoxic conditions, HIF-1*α*, has been used in previous studies to evaluate IR injury and has been proven to protect myocardial cells from IR injury [29]. The protein also has a protective effect on fibroblasts [30]. Expression of HIF-1*α* does not increase in muscle flaps during ischemia [31], attributed to the already high levels of HIF-1*α* in muscle tissue. In contrast, the HIF-1*α* expression in our study was statistically higher after IR injury in all zones of muscle and skin tissues, indicating that the tissue tolerance against ischemia increased. HIF-1 can then decrease ROS production upon reperfusion by altering mitochondrial metabolism, increasing adenosine levels, and also inducing small levels of ROS production that then elicit an antioxidant response [32].

The present study also confirmed that HIF-1*α* stimulates VEGF production [33]. This was particularly evident in the BoTA group, suggesting that BoTA's protective effect against IR injury depends on increasing angiogenesis via the HIF-1*α*/VEGF pathway. In our study, we used CD34, a neoangiogenesis-associated cell surface protein, as a marker for the extent of protection that a tissue had against IR injury. The CD34 expression was statistically higher in the BoTA group, both in skin and in muscle.

One interesting finding in our study was the significantly increased expression of NF-kB in the BoTA group. The NF-kB protein is involved in the regulation of migration, proliferation, and/or survival of endothelial cells [34]. The well-known NF-kB pathway transcribes many inflammatory molecules known to be associated with neutrophil accumulation. Inflammatory pathways, including the NF-kB pathway, are activated by ROS and are closely related to IR injury in skeletal muscles [35]. Although there was some thought that NF-kB activation could lead to a decrease in extracellular matrix degradation capacity, in turn leading to impaired angiogenesis [34], NF-kB transcription factors have been found to be effectors of the Rho family, including molecules such as the GTP-binding protein RAC1, during inflammation [19, 36]. As the Rho family is involved in a critical signaling pathway in angiogenesis, the results of our current study require further study to elucidate this apparently novel role played by NF-kB in IR injury pathogenesis.

In our study, we used a superior epigastric artery-based axial flap. We applied BoTA to different microcirculation zones to evaluate BoTA's local effect on different microvascular circulation patterns. In order to eliminate the vasodilatation effect of BoTA on the main pedicle and evaluate BoTA's local mechanism in an axial flap, we injected the toxin subcutaneously, not in the muscle.

We calculated the viable areas of flaps as a predictive factor for IR injury. A 6 × 1.5 cm, rectangular, musculocutaneous flap was designed to evaluate the effect of BoTA on flap viability after IR injury. As expected, the local BoTA application significantly decreased the necrotic flap area when compared to that in the control group. This result agreed with previous studies on the effects of BoTA in skin flaps [15–18]. We think that BoTA may protect against IR injury at the cellular level, even beyond its vasodilatation and increased flap circulation capabilities. More studies are needed to elucidate the definite mechanism. Although the presence of increased gene expression does not translate into increased protein expression and the increased gene expression may not be clinically relevant to the fact that BoTA pretreatment improved flap survival, we think local preischemic preconditioning with BoTA attenuated flap necrosis following many flap surgeries pertaining to pedicle twisting based on our findings. However, local BoTA preconditioning has clinical limitations because it requires further investigation of adequate dosage and interval.

The plastic tourniquet that we used was constantly adopted without any difficulty (Supplement Figure 1, in Supplementary Material available online at https://doi.org/10.1155/2017/1074178). As this tourniquet does not allow being loosened after tightening, once we tighten the pedicle as much as possible with this tourniquet, the experiment can be very reliable. We believe that this is very easy to control the pressure evenly compared to previously well-known suture tie method.

Our present study also has some limitations. The major limitation of the current study was that IR injury is mainly a problem encountered in free flap surgery or replantation but not in pedicled flap surgery. Therefore, our experimental model, the elevation of a pedicled flap without microanastomosis, does not seem like an ideal model. Secondly, the number of animals is relatively small which limits the conclusions that could be drawn and future studies are warranted to elucidate the delay effect of BoTA on musculocutaneous flap in a clinical setting. Third, we did not conduct any pathologic exams in a current study; future works should be expanded to histology and immunohistochemistry to determine the effects on the cellular level as well.

## 5. Conclusions

In this study, we found that local BoTA preconditioning yielded significant protection against IR injury in a rat model.

## Supplementary Material

The plastic tourniquet used in our study.
