# Supplementary figures and images for: The Effect of Botulinum Toxin A on Ischemia-Reperfusion Injury in a Rat Model
Source: Biomed Res Int. 2017 May 15;2017:1074178. doi: 10.1155/2017/1074178 (PMC5447266; doi:10.1155/2017/1074178)

Supplement figure 1

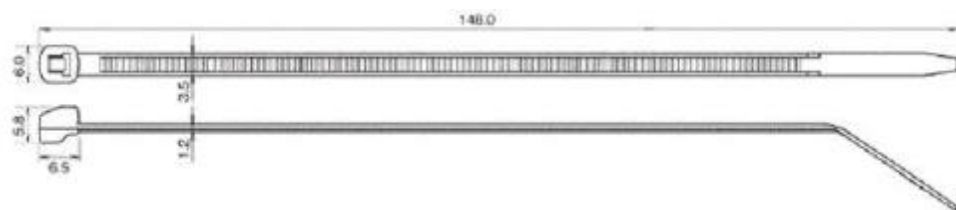

Supplement: Supplementary file 1 — The plastic tourniquet used in our study. [file 1074178.f1.pdf]
